# Selection of Medical Students

**Published:** 1945

**Authors:** Frank Bodman

**Affiliations:** Acting Director: Bristol Child Guidance Clinic. Psychiatrist, Somerset County Council


### SELECTION OF MEDICAL STUDENTS

BY

FRANK BODMAN, M.D.,

*Acting Director: Bristol Child Guidance Clinic.*
*Psychiatrist, Somerset County Council.*

(Read to the Study Group, Bristol Division, B.M.A., 16th February, 1945.)

---

In considering the question of selection of medical students, we must take into consideration the greatly increased number of doctors required to implement a national medical service. We must also bear in mind the implication of Mr. Butler's Education Act. More boys and girls will receive a secondary education, and more adolescents will see their ambition of becoming a doctor become a practical possibility, particularly as further grants to universities, a million pounds for medical education, may make the education of these boys a practical proposition.

Why should selection be necessary if increased numbers are wanted ? Why not take them all ? On purely economic grounds it would be wasteful to spend State money on training unsuitable candidates. From the point of view of the candidates themselves, it would be unfair to permit them to start on a course of training taking several years if they were for various reasons unfitted for the profession. But the main argument would be that it was contrary to the public interest to enlist in the profession men and women who were incapable of becoming " proper " doctors.

What are the motives, the incentives which influence a student to take up medicine ? The obvious ones are the conscious ones, but such choices are also to a large extent determined by unconscious motives.

Of the obvious motives there are :—

1. Security. Although the monetary rewards are not comparable with those of many other walks of life, they are sufficiently good to be attractive, particularly in times of depression.

2. Social prestige, particularly to the clever boy who comes from a skilled worker's home, the status of the doctor makes some appeal. He drives about in a car, has a bigger house than most of his patients, and is looked up to some extent as a leader.

3. Curiosity—scientific interest. The workings of the human organism are a tremendous source of interest to intelligent laymen, and a desire to know more about them is often a quite compelling drive to read medicine.

4. Humanitarian drives. The conscious wish to be of service to fellow human beings is quite a powerful drive in a small percentage of cases. This is a factor perhaps more commonly observed in women.

5. The wish to follow in the father's footsteps, more rarely in the mother's. This is not a very common factor—less common than we realize perhaps. Probably not more than 20 per cent. of doctors' sons take up medicine.

These perhaps are the more common overt drives, the drives admitted in consciousness. But combined with these drives are often drives which are unconscious in origin, and not so easily admitted. Perhaps the more common unconscious drive is a will to power. The doctor because of his special knowledge and his experience is bound to be looked up to as an authority. In a civilization where many people in peace time have never witnessed a birth, or seen a corpse before they are forty, the doctor with his long experience of these fundamental events is looked up to as an authority in times of crisis even if he is disparaged in between times. And those individuals with a will to power, conscious or unconscious, are bound to be attracted to a profession that carries the keys of life and death. Their superior knowledge, their extensive experience, places them at an advantage over the layman.

Another quite frequent unconscious drive is the need to come to terms with death. It is not uncommon for the young boy or girl to decide to take up medicine after the serious illness or death of one or other parent. This threat to their security must be mastered in the future. They must arm themselves with the knowledge which will ward off this threat, or this loss. Sometimes it is not the threat of death or actual bereavement of a parent ; it may be the fear of death as a personal threat that unconsciously influences the adolescent to take up medicine.

Of course, in most instances the decision to be a doctor is the resultant of a number of drives, conscious and unconscious ; but it can be well appreciated that the best doctors will not be those who have been mainly driven by factors which are prestige factors—overt desire for improved social status—or unconscious desires to dominate others.

We have then so much raw material, ambitious, scientific, humanitarian, hero-worshipping, looking-for-a-safe-job, wanting-to-dominate, unconsciously-afraid-of-death.

And we hope to turn this raw material into what ? We expect the medical student not only to acquire the necessary professional knowledge and skill, but we also expect him to acquire the professional attitudes—and here we may pause to ask ourselves what is a professional attitude ? It involves a respect for other people as human beings, as persons with their own rights. It involves a responsibility for the work done for those people. The job must come first. One can recognize the professional attitude not only amongst the learned professions : one can recognize it in policemen, wardens, shelter-marshals. Their responsibility for other people comes before personal considerations. Choices must be made, risks must be taken, which involve the health and lives of other persons ; but the responsibility for those choices, for those risks, cannot be pushed off on to others—they are the doctor's responsibility. The Bible has a word for it : " He that is a hireling and not a shepherd, whose own the sheep are not, beholdeth the wolf coming and fleeth, and the wolf snatcheth them and scattereth them ; he fleeth because he is a hireling and careth not for the sheep."

So that there are two main objectives to be attained : (1) the professional skills and knowledge, (2) the professional attitude. The first is a matter largely of intelligence supported by special factors. The second is a question of temperament and personality.

The medical student :—

1. Must have ability to use all the senses in keen observation, with a capacity for a new range of appreciation of what is significant.

2. Should be able to express in accurate words what is observed, and the meaning of his observations.

3. Needs a flexible mind, capable of associating quickly and easily ; he must be able to make comparisons easily ; he must sense the general in a mass of particulars, and apply general principles to particular instances. That is to say, he must be able to synthesize as well as analyze ideas and concepts.

Those are the intellectual requirements.

Then as far as personality goes :—

1. He must be able to identity with a variety of people, in many situations—be all things to all men—without losing his intellectual integrity.

2. He must be able to feel appropriately to the situation without losing the ability to see it in perspective so that he can help the patient who is so submerged in his pain, suffering, and anxiety, that he cannot see beyond it.

3. He must be able to express warmth of feeling in appropriate ways, if he is to create confidence in the doctor-patient relationship.

You will say, this is a tall order, and how are these things to be judged in an adolescent ? It is true there are grave difficulties here, for the medical student at the beginning of his career is economically —often emotionally—still dependent on his family, and if he is to mature must become independent. Moreover, biologically he is still in the process of making adjustments to the opposite sex, and has for the most part not achieved a more than partially mature attitude. Both these important processes—detachment from the family and adaptation to the opposite sex—are bound to have a disturbing effect on his emotional stability, so that such a time is not the optimum time for an assessment.

For the intellectual factors in acquiring professional knowledge and skill, it is possible now to give intelligence tests which can rule out those whose intellectual capacities are such that they cannot achieve the necessary standards : (the base line will probably be found at an I.Q. of 115). Probably the Matrices Test is the best test for conceptual thinking.

But for the personal and temperamental factors there can be no standardized test at the moment. The range of medicine from the laboratory to the bedside is so great that job-analysis is not feasible in the present state of our knowledge, and in any case we are unlikely to reach absolute standards ; we can only achieve relative ones.

The examiner will always be limited to some extent by his unconscious reactions to the candidate, and his own unconscious attitudes to the profession. These unconscious factors, because they are unconscious, are beyond control. But in the really mature individual, there are smaller and smaller biases, and the relative standards approximate closer to the ideal. These personal and temperamental factors, therefore, can only be assessed by the technique of the interview. But the interview is a very skilled technique, which requires special experience and special attitudes. There are not many people qualified to do this job, and personal qualifications are probably more important than academic status. It does not necessarily follow that a dean or a professor of medicine would make the best interviewer. It required someone with the broadest possible experience of life, and a special experience of watching and teaching students to assess the still only partly developed capacities, and gauge how far the personality of the student will be developed by the experiences of life encountered in his training. But some of his pointers will be the eagerness for new experience. the family tradition of work, the estimated capacity for professional feeling, the degree of self-discipline attainable, and the amount of responsibility likely to be developed.

### BIBLIOGRAPHY.

B. C. Reynolds. *Learning and Teaching in the Practice of Social Work*, New York. (1942).

Edholm, O. G., and Gibson, Q. H. (1944). *Lancet*, ii. 2942–96, ii. 549.

Moran (1944). *Lancet*, ii. 277.

